# Supplementary material for: Egg Protein Compositions over Embryonic Development in Haemaphysalis hystricis Ticks
Source: Animals (Basel). 2024 Nov 30;14(23):3466. doi: 10.3390/ani14233466 (PMC11640286; doi:10.3390/ani14233466)
Supplement: Supplementary file 1 [file animals-14-03466-s001.zip › Supplementary-Table S1.pdf]

Supplementary Materials

Table S1. Top four proteins identified from the LC-MS/MS analysis of protein bands from day one egg samples

Band	Transcripts ID in PRJNA1168713	iBAQ	Precent (%)	**Alignment Entry and overview**	∑n
1	Cluster-17602.36441	7.89E+07	0.42	A0A1Z5LGC3, Actin 1, Ornithodoros moubata	187281365.7
	Cluster-17602.43418	5.61E+07	0.30	A0A8X8MG03, Vitellin-a, Haemaphysalis flava	187281365.7
	Cluster-17602.25911	1.01E+07	0.05	A0A411G179, Vitellogenin, Haemaphysalis flava	187281365.7
	Cluster-17602.35798	1.23E+07	0.07	A0A131XJZ2, Tubulin beta chain, Hyalomma excavatum	187281365.7
2	Cluster-17602.43418	3.12E+08	0.82	A0A8X8MG03, Vitellin-a, Haemaphysalis flava	377948530
	Cluster-17602.36441	1.84E+07	0.05	A0A1Z5LGC3, Actin 1, Ornithodoros moubata	377948530
	Cluster-17602.25911	1.70E+07	0.04	A0A411G179, Vitellogenin, Haemaphysalis flava	377948530
	Cluster-17602.35895	1.58E+07	0.04	Microtubule associated complex, Rhipicephalus zambeziensis	377948530
3	Cluster-17602.25911	2.85E+09	0.67	A0A411G179, Vitellogenin, Haemaphysalis flava	4232957045
	Cluster-17602.43418	8.70E+08	0.21	A0A8X8MG03, Vitellin-a, Haemaphysalis flava	4232957045
	Cluster-17602.16459	3.44E+08	0.08	A0A023FZQ2, erine/threonine protein kinase, Amblyomma parvum	4232957045
	Cluster-17602.35680	3.43E+07	0.01	A0A131XLQ7, Histone H2B, Hyalomma excavatum	4232957045
4	Cluster-17602.43418	3.57E+09	0.73	A0A8X8MG03, Vitellin-a, Haemaphysalis flava	4903290137
	Cluster-14619.0	6.44E+08	0.13	A0A1E1X9Y4, alpha-L-fucosidase, Amblyomma aureolatum	4903290137
	Cluster-17602.25911	5.23E+08	0.11	A0A411G179, Vitellogenin, Haemaphysalis flava	4903290137
	Cluster-17602.60258	5.76E+07	0.01	L7LU83, tick transposon, Rhipicephalus pulchellus	4903290137
5	Cluster-17602.25911	3.25E+09	0.53	A0A411G179, Vitellogenin, Haemaphysalis flava	6143419827
	Cluster-17602.43418	2.82E+09	0.46	A0A8X8MG03, Vitellin-a, Haemaphysalis flava	6142443887
	Cluster-17602.34770	9.14E+06	0.00	B1B544, Vitellogenin-2, Haemaphysalis longicornis	6143419827
	Cluster-17602.30102	8.51E+06	0.00	A0A023GL99, Glucose-6-phosphate isomerase, Amblyomma triste	6143419827

6	Cluster-17602.25911	3.40E+09	0.87	A0A411G179，Vitellogenin，Haemaphysalis flava	3899151182
	Cluster-17602.43418	2.75E+08	0.07	A0A8X8MG03，Vitellin-a，Haemaphysalis flava	3899151182
	Cluster-17602.42323	5.21E+07	0.01	A0A6M2CIQ7, Alpha-amylase, Rhipicephalus microplus	3899151182
	Cluster-17602.6001	4.38E+07	0.01	？mucin need to be confirmed	3899151182
7	Cluster-17602.15229	1.83E+09	0.81	A0A6M2CKA2， Yolk cathepsin，Rhipicephalus microplus	2267489780
	Cluster-17602.43418	1.73E+08	0.08	A0A8X8MG03，Vitellin-a，Haemaphysalis flava	2267489780
	Cluster-17602.25911	1.10E+08	0.05	A0A411G179，Vitellogenin，Haemaphysalis flava	2267489780
	Cluster-17602.31952	1.09E+08	0.05	A0A5P8H6S1，Serpin-a，Haemaphysalis longicornis	2267489780
8	Cluster-17602.33592	4.31E+08	0.69	A0A6M2CV56, cathepsin d isoform 1 protein, Rhipicephalus microplus	620750086.6
	Cluster-17602.25911	1.27E+08	0.20	A0A411G179，Vitellogenin，Haemaphysalis flava	620750086.6
	Cluster-17602.50192	3.43E+07	0.06	A0A6M2CHZ1，Heme-binding aspartic proteinase，Rhipicephalus microplus	620085106.6
	Cluster-17602.43418	1.90E+07	0.03	A0A8X8MG03，Vitellin-a，Haemaphysalis flava	620750086.6
9	Cluster-17602.25911	4.92E+08	0.71	A0A411G179，Vitellogenin，Haemaphysalis flava	689465860
	Cluster-17602.43418	9.02E+07	0.13	A0A8X8MG03，Vitellin-a，Haemaphysalis flava	687539360
	Cluster-17602.33592	5.37E+07	0.08	A0A6M2CV56, cathepsin d isoform 1 protein, Rhipicephalus microplus	689465860
	Cluster-17602.34926	1.38E+07	0.02	A0A1E1XVN0, Histone H4, Amblyomma sculptum	689465860
10	Cluster-17602.30986	1.46E+07	0.61	A0A034WTW0，Kunitz domain-containing protein 1，Rhipicephalus microplus	23857906
	Cluster-17602.55588	1.36E+06	0.06	A0A9J6FIL7, Uncharacterized protein, Haemaphysalis longicornis	22861266
	Cluster-17602.29432	1.23E+06	0.05	A0A5B9BYB0，Cysteine-rich protein，Haemaphysalis flava	23857906
	Cluster-17602.35680	9.33E+05	0.04	A0A131XLQ7, Histone H2B, Hyalomma excavatum	23857906

11	Cluster-17602.30986	5.30E+07	0.64	A0A034WTW0，Kunitz domain-containing protein 1，Rhipicephalus microplus	82347646
	Cluster-17602.43418	8.75E+06	0.11	A0A8X8MG03，Vitellin-a，Haemaphysalis flava	77018746
	Cluster-11893.0	4.43E+06	0.05	A0A034WTW4，Serine proteinase inhibitor，Rhipicephalus microplus	82347646
	Cluster-17602.29432	2.97E+06	0.04	A0A5B9BYB0，Cysteine-rich protein，Haemaphysalis flava	82347646
12	Cluster-17602.25911	1.32E+08	0.60	A0A411G179，Vitellogenin，Haemaphysalis flava	219499030
	Cluster-11893.0	2.10E+07	0.10	A0A034WTW4，Serine proteinase inhibitor，Rhipicephalus microplus	219499030
	Cluster-17602.30986	2.03E+07	0.09	A0A034WTW0，Kunitz domain-containing protein 1，Rhipicephalus microplus	219499030
	Cluster-17602.34926	1.56E+07	0.08	A0A1E1XVN0, Histone H4, Amblyomma sculptum	197965000
