# Supplementary material for: Egg Protein Compositions over Embryonic Development in Haemaphysalis hystricis Ticks
Source: Animals (Basel). 2024 Nov 30;14(23):3466. doi: 10.3390/ani14233466 (PMC11640286; doi:10.3390/ani14233466)
Supplement: Supplementary file 1 [file animals-14-03466-s001.zip › Supplementary-Table S2.pdf]

Table S2. High-confidence transtripts identified in eggs of *H. hystricis* on day-one incubation.

No	Transcripts ID in PRJNA1168713	Alignment Entry and overview	E value	Score	Identity (%)	iBAQ (× 10^6)
TRANS-PORT-ERS *	Cluster-17602.43418	A0A8X8MG03, Vitellin-a, *Haemaphysalis flava*	0	9,170	93.30%	15110
	Cluster-17602.25911	A0A411G179, Vitellogenin, *Haemaphysalis flava*	0	8,943	87.50%	13894
	Cluster-17602.45411+Cluster-17602.36443	A0A346JM05, Vitellogenin-1, *Haemaphysalis flava*	9.90E-115	932	95.00%	313.938
	Cluster-17602.28618	A0A346JM06, Vitellogenin-2, *Haemaphysalis flava*	0	2,573	86.50%	31.164
	Cluster-17602.36611+Cluster-17602.34770	B1B544, Vitellogenin-2, *Haemaphysalis longicornis*	0	5,815	95.10%	555.77
	Cluster-17602.36550	A0A346JM08, Vitellogenin-B, *Haemaphysalis flava*	0	14,976	89.20%	12.138
	Cluster-17602.36154	M5AYG7, Ferritin, *Haemaphysalis longicornis*	5.70E-138	999	99.00%	51.945
	Cluster-17602.26911	A0A6G5A4V7, Lipocalin, *Rhipicephalus microplus*	9.50E-42	364	41.80%	12.347
	Cluster-17602.9099	G3MQN9, Lipocalin-5 1, *Amblyomma maculatum*	2.60E-58	473	50.30%	177.98
	Cluster-14884.0	A0A224YMV4, Lipocalin, *Rhipicephalus zambeziensis*	5.00E-64	511	55.10%	7.1101
	Cluster-17602.12024	A0A131Z3F4, Lipocalin, *Rhipicephalus appendiculatus*	6.80E-49	409	49.10%	37.255
	Cluster-17602.18227	A0A224YBF9, Lipocalin, Rhipicephalus zambeziensis	4.10E-71	556	55.20%	17.007
	Cluster-17602.28561	A0A034WU22, Lipocalin 1, *Rhipicephalus microplus*	3.30E-65	519	53.60%	12.206
	Cluster-16489.0	A0A023GAE6, Lipocalin-1 1, *Amblyomma triste*	8.80E-29	270	46.80%	34.798
	Cluster-18314.0	A0A023G979, Lipocalin-2 1, *Amblyomma triste*	4.90E-84	642	60.90%	18.846
	Cluster-17602.37869	A0A023GCN1, Lipocalin-5 1, *Amblyomma triste*	2.20E-65	518	51.40%	6.8753
	Cluster-17602.4060	A0A0C9R5X8, Lipocalin-5 1, *Amblyomma americanum*	1.90E-38	341	40.00%	30.778
	Cluster-17602.14012	A0A023GA06, Lipocalin-8 1, *Amblyomma triste*	5.50E-65	514	56.70%	97.572
	Cluster-17602.11248	A0A034WWH8, Lipocalin 22, *Rhipicephalus microplus*	1.60E-78	606	65.10%	28.119
	Cluster-17602.41506	A0A1E1XBG7, Lipoprotein amino terminal region, *Amblyomma aureolatum*	0	8,923	70.30%	18.036
EN-ZYMES	Cluster-17602.15229	A0A6M2CKA2, Yolk cathepsin, *Rhipicephalus microplus*	0	1,372	66.80%	7670.2
	Cluster-17602.34900	A0A224YLY0, Midgut cysteine proteinase 2, *Rhipicephalus zambeziensis*	7.70E-86	683	78.00%	63.219
	Cluster-17602.35763	A0A1E1XAU4, Midgut cysteine proteinase, *Amblyomma aureolatum*	6.40E-114	871	92.40%	66.592
	Cluster-17602.39009	A0A1E1XAU4, Midgut cysteine proteinase, *Amblyomma aureolatum*	3.80E-63	525	84.20%	69.482
	Cluster-17602.36868	A0A411HE54, Cathepsin L-like cysteine protein, *Haemaphysalis flava*	0	1,706	92.50%	53.408
	Cluster-17602.33992	A0A6M2CJ69, Aspartic protease, *Rhipicephalus microplus*	8.50E-172	1,262	62.80%	29.304
	Cluster-17602.50192	A0A6M2CHZ1, Heme-binding asparlic proteinase, *Rhipicephalus microplus*	5.10E-128	957	74.60%	534.52
	Cluster-11877.0	A0A8K1PH81, Heme-binding asparlic proteinase, *Rhipicephalus microplus*	0	1,347	67.50%	1403.2
	Cluster-17602.35688	Q2WFX6, Aspartic protease, *Haemaphysalis longicornis*	0	2,012	98.00%	4.7188
	Cluster-17602.1417	A0A6M2CGL6, Cathepsin D, *Rhipicephalus microplus*	1.20E-89	715	44.60%	74.931
	Cluster-18960.0	A0A131YNC8, Cathepsin D, *Rhipicephalus appendiculatus*	3.60E-67	548	53.50%	156.23

	Cluster-17602.21941	A0A131XWE4, Neurotrypsin, *Ixodes ricinus*	0	3,446	53.80%	0.98982
	Cluster-17602.36343	A0A023GPG6, Dipeptidyl peptidase 1, *Amblyomma triste*	0	2,131	87.00%	4.3299
	Cluster-17602.37507	A0A224YPI2, Phospholipase B-like, *Rhipicephalus zambeziensis*	0	1,702	83.00%	18.915
	Cluster-17602.27833	A0A6P7UWU0, Carboxypeptidase, *Ixodes scapularis*	0	1,444	58.10%	11.616
	Cluster-17602.32606	G3MF47, Alpha-amylase , *Amblyomma maculatum*	1.60E-131	999	67.80%	317.52
	Cluster-17602.13444	A0A131YTV0, Alpha-galactosidase , *Rhipicephalus appendiculatus*	0	1,879	72.40%	2.1796
	Cluster-17602.36136	A0A6G6CUG5, Alpha-mannosidase protein, *Haemaphysalis flava*	0	3,789	91.30%	1.1726
	Cluster-17602.34511	A0A6M2CTD4, ATP synthase subunit beta, *Rhipicephalus microplus*	0	2,609	96.70%	0.34924
	Cluster-17602.37399	A0A1E1X7H4, Glucosidase ii catalytic alpha subunit, *Amblyomma aureolatum*	0	1522	70.80%	82.027
	Cluster-17602.35564	A0A224YJ06, Peptidyl-prolyl cis-trans isomerase, *Rhipicephalus zambeziensis*	7.40E-119	873	84.60%	44.626
	Cluster-17602.35954	A0A1E1XCW7, Glutathione s-transferase, *Amblyomma aureolatum*	3.90E-123	906	78.30%	26.359
	Cluster-17602.38580	A0A023FXL8, Aldehyde dehydrogenase, *Amblyomma parvum*	0	1,906	90.80%	0.84185
	Cluster-17602.11286	B7QAW0, Glucose dehydrogenase, putative, *Ixodes scapularis*	0	1,970	64.10%	49.033
	Cluster-17602.37559	A0A7U0REX2, Glyceraldehyde-3-phosphate dehydrogenase, *Haemaphysalis longicornis*	0	1,685	99.40%	3.0241
	Cluster-17602.16862	A0A023FN97, Catalase, *Amblyomma cajennense*	1.40E-156	1,160	73.90%	45.774
	Cluster-17602.16856	A0A6M2CJC9, Catalase, *Rhipicephalus microplus*	7.70E-143	1,085	73.20%	64.124
	Cluster-17602.35118	A0A146J914, Peroxiredoxin, *Haemaphysalis longicornis*	5.60E-128	931	96.60%	24.008
IMMUNITY-RELATED PROTEIN	Cluster-17602.7590	Q86LE5, Antimicrobial peptide microplusin, *Rhipicephalus microplus*	6.80E-14	171	39.40%	30.002
	Cluster-17602.7506	B7PQ34, Peroxinectin, *Ixodes scapularis*	0	1,668	56.30%	12.495
	Cluster-17602.36041	A0A023FNM2, Alpha-2-macroglobulin-like protein, *Amblyomma cajennense*	0	6,492	80.30%	26.076
	Cluster-17602.29432	A0A5B9BYB0, Cysteine-rich protein, *Haemaphysalis flava*	3.10E-110	810	86.70%	1112.7
	Cluster-17602.34079	A0A1E1X522, Ficolin, *Amblyomma aureolatum*	0	1424	83.30%	7.8369
	Cluster-17602.8619	A0A6M2D6P5, His-rich 1 fat body overexpressed, *Rhipicephalus microplus*	5.10E-17	188	38.10%	1499.2
	Cluster-17602.7590	A0A6M2D6P5, His-rich 1 fat body overexpressed, *Rhipicephalus microplus*	1.80E-17	191	40.20%	2158.1
PROTEANASE INHIBITORS	Cluster-17602.39215	A0A5P8H6S1, Serpin-a, *Haemaphysalis longicornis*	0	1,872	93.70%	22.442
	Cluster-17602.36491	A0A5P8H6S1, Serpin-a, *Haemaphysalis longicornis*	8.50E-167	1,231	63.20%	14.346
	Cluster-17602.35298	A0A5P8H6S1, Serpin-a, *Haemaphysalis longicornis*	6.30E-169	1,245	62.70%	10.889
	Cluster-17602.31952	A0A5P8H6S1, Serpin-a, *Haemaphysalis longicornis*	7.40E-158	1,172	61.20%	1180.6
	Cluster-9037.0	A0A5P8H6S1, Serpin-a, *Haemaphysalis longicornis*	2.10E-150	1,123	55.90%	3.4807
	Cluster-17602.35498	Q75Q63, Serpin-2, *Haemaphysalis longicornis*	0	1,933	95.70%	18.179
	Cluster-18187.0	A0A023GN63, Serpins 9, *Amblyomma triste*	2.90E-27	271	54.20%	107
	Cluster-11893.0	A0A034WTW4, Serine proteinase inhibitor, *Rhipicephalus microplus*	1.10E-29	269	56.80%	4204
	Cluster-21278.0	A0A6M2CIH3, Kunitz domain-containing protein 1, *Rhipicephalus microplus*	8.50E-100	799	42.90%	209.5
	Cluster-22020.0	A0A034WTW0, Kunitz domain-containing protein 1, *Rhipicephalus microplus*	2.90E-106	854	45.90%	13.985
	Cluster-17602.19391	A0A034WTW0, Kunitz domain-containing protein 1, *Rhipicephalus microplus*	0	1,483	41.50%	396.61
	Cluster-17602.29821	A0A6M2CIH3, Kunitz domain-containing protein 1, *Rhipicephalus microplus*	8.10E-67	569	37.00%	65.326

	Cluster-17602.30986	A0A034WTW0, Kunitz domain-containing protein 1 , *Rhipicephalus microplus*	0	1,832	34.50%	4493.1
	Cluster-17602.36134	A0A8F1NJE0, Neutrophil elastase inhibitor, *Haemaphysalis flava*	4.60E-32	294	56.40%	539.96
	Cluster-17602.10962	A0A8F1NJE0, Neutrophil elastase inhibitor, *Haemaphysalis flava*	5.70E-30	270	53.50%	1627.7
	Cluster-17602.33042	A0A8F1NJE0, Neutrophil elastase inhibitor, *Haemaphysalis flava*	6.80E-63	492	90.20%	63.93
	Cluster-17602.18555	A0A8F1NJE0, Neutrophil elastase inhibitor, *Haemaphysalis flava*	7.40E-49	394	73.50%	12488
	Cluster-17602.35730	A0A8F1NJE0, Neutrophil elastase inhibitor, *Haemaphysalis flava*	6.40E-47	392	72.50%	11522
	Cluster-17602.34822	A0A8F1NJF7, Neutrophil elastase inhibitor, *Haemaphysalis flava*	2.50E-05	107	25.20%	103.78
	Cluster-17602.41892	A0A1E1XRH0, Rab GDP dissociation inhibitor, *Amblyomma sculptum*	0	1,968	98.20%	3.0926
HEAT SHOCK PRO-TEINS	Cluster-17602.35965	E4W3Z2, Heat shock 70 kDa protein 5, *Haemaphysalis longicornis*	0	3,318	99.20%	12.19
	Cluster-17602.36516	A0A097A1J8, Heat shock 70 kDa protein 8, *Haemaphysalis flava*	0	3281	99.10%	5.7437
	Cluster-17602.39985	A0A2D1C0A7, Heat shock protein 83, *Haemaphysalis flava*	0	1,752	98.80%	7.1571
CYTO-SKELE-TAL PRO-TEINS	Cluster-17602.37337	C8YL72, Calreticulin, *Haemaphysalis longicornis*	0	2,238	98.00%	2.3001
	Cluster-17602.36441	A0A1Z5LGC3, Actin 1, *Ornithodoros moubata*	0	1,961	99.70%	298.22
	Cluster-17602.36310	A0A131XLQ1, Tubulin alpha chain, *Hyalomma excavatum*	0	2,385	99.10%	9.8667
	Cluster-17602.33432	A0A293MD80, Tubulin beta chain, *Ornithodoros erraticus*	0	1,629	98.10%	7.9497
	Cluster-17602.35092	A0A1E1X761, Microtubule associated complex, *Amblyomma aureolatum*	2.60E-98	828	53.30%	62.688
SE-CRETED PEP-TIDES	Cluster-17602.45999	A0A023GBD7, Secreted protein, *Amblyomma triste*	7.90E-137	1011	82.60%	18.059
	Cluster-17602.33713	L7M9B9, amblyomma 40-33 family member, *Rhipicephalus pulchellus*	3.30E-78	603	76.50%	52.134
	Cluster-17602.23387	A0A023GN50, Secreted protein, *Amblyomma triste*	0	1,340	82.00%	3.5525
	Cluster-17602.36822	A0A023GB40, Secreted protein, *Amblyomma triste*	4.90E-112	834	74.30%	38.598
	Cluster-17602.23292	A0A6B0UWG0, Conserved secreted protein, *Ixodes ricinus*	4.50E-45	379	44.30%	207.47
	Cluster-17602.34763	A0A023FC77, Secreted papa repeat protein, *Amblyomma cajennense*	2.10E-08	138	41.10%	4.3277
	Cluster-17602.36297	A0A1E1XNR5, Conserved secreted protein, *Amblyomma sculptum*	1.40E-60	485	60.30%	177.36
	Cluster-17805.0	B7QGW1, Secreted protein, *Ixodes scapularis*	2.40E-19	207	42.30%	1968.3
	Cluster-17602.37290	A0A1E1XM23, Secreted protein, *Amblyomma sculptum*	4.4e-136	1,013	66.80%	9.4752
NCHAR-ACTER-IZED PRO-TEIN	Cluster-17602.37876	A0A224YT51, Gov platelet alloantigens, *Rhipicephalus zambeziensis*	0	5163	62.10%	19.55
	Cluster-17602.27619	A0A131YW52, Rhodanese domain-containing protein, *Rhipicephalus appendiculatus*	0	3,398	38.80%	36.9
	Cluster-17602.34802	A0A1E1X1G9, Secreted salivary protein, *Amblyomma aureolatum*	3.80E-38	327	59.40%	35.421
	Cluster-17602.36732	G3MS15, Amb_all domain-containing protein, *Amblyomma maculatum*	1.20E-63	501	85.50%	6.4652
	Cluster-17602.12808	A0A182TMR5, Cuticular protein, *Anopheles melas*	2.00E-15	194	29.70%	1436.7
	Cluster-12057.0	Q202J4, Dermonecrotic toxin SPH, *Ixodes scapularis*	9.60E-36	333	44.90%	2080.2
	Cluster-17602.47512	A0A6M2CWG2, Sphingomyelinase d-like protein, *Rhipicephalus microplus*	5.70E-122	916	58.10%	8.7084
	Cluster-17602.39905	A0A6M2E4I3, Cytochrome b5, *Amblyomma tuberculatum*	1.90E-58	461	95.7%%	14.56
	Cluster-17602.25769	A0A1E1XCC5, Membrane glycoprotein lig-1, *Amblyomma aureolatum*,	0	1,564	69.40%	7.5152
	Cluster-17602.34460	A0A224Z7V2, Serum amyloid A protein, *Rhipicephalus zambeziensis*	2.50E-74	571	70.70%	29.119

Cluster-17602.49041	A0A131YDT0, Cofilin, *Rhipicephalus appendiculatus*	4.80E-98	726	95.90%	8.0561
Cluster-17602.35895	A0A224YYX5, Microtubule associated complex, *Rhipicephalus zambeziensis*	0	16,347	68.90%	219.54
Cluster-17602.36724	A0A023FLP4, Elongation factor 1-alpha, *Amblyomma cajennense*	0	2,404	98.90%	4.8041
Cluster-17602.38192	G3MIH5, SCP domain-containing protein, *Amblyomma maculatum*	5.10E-144	1070	69.00%	42.639
Cluster-17602.36289	A0A224YW71, Polyubiquitin-B, *Rhipicephalus zambeziensis*	4.30E-67	517	99.00%	6.1271
Cluster-17602.33890	G3MQ03, 14_3_3 domain-containing protein, *Amblyomma maculatum*	2.40E-170	1,221	100.00%	13.992

* iBAQ: intensity-based absolute quantification; .
